# Ameliorating diabetes-associated atherosclerosis and diabetic nephropathy through modulation of soluble guanylate cyclase

**DOI:** 10.3389/fcvm.2023.1220095

**Published:** 2023-07-12

**Authors:** Arpeeta Sharma, Judy Choi, Lachlan Sim, Abhiroop Dey, Muthukumar Mohan, Phillip Kantharidis, Lisa Dietz, Peter Sandner, Judy B. de Haan

**Affiliations:** ^1^ Cardiovascular Inflammation and Redox Biology Laboratory, Baker Heart and Diabetes Institute, Melbourne, VIC, Australia; ^2^ Department of Diabetes, Monash University, Central Clinical School, Melbourne, VIC, Australia; ^3^ Pharmaceuticals Research and Development, Bayer AG, Wuppertal, Germany; ^4^ Institute of Pharmacology, Hannover Medical School, Hanover, Germany; ^5^ Baker Department of Cardiometabolic Health, University of Melbourne, Parkville, VIC, Australia; ^6^ Department Immunology and Pathology, Central Clinical School, Monash University, Melbourne, VIC, Australia; ^7^ Baker Department Cardiovascular Research, Translation and Implementation, La Trobe University, Melbourne, VIC, Australia; ^8^ Faculty of Science, Engineering and Technology, Swinburne University, Melbourne, VIC, Australia

**Keywords:** soluble guanylate cyclase, type 2 diabetes, atherosclerosis, endothelial dysfunction, nitric oxide, inflammation, oxidative stress

## Abstract

Diabetes mellitus (DM) is an independent risk factor for micro- and macrovascular complications such as nephropathy and atherosclerosis respectively, which are the major causes of premature morbidity and mortality in Type 1 and Type 2 diabetic patients. Endothelial dysfunction is the critical first step of vascular disease and is characterized by reduced bioavailability of the essential endothelial vasodilator, nitric oxide (NO), coupled with an elevation in inflammation and oxidative stress. A novel pathway to bolster NO activity is to upregulate soluble guanylate cyclase (sGC), an enzyme responsible for mediating the protective actions of NO. Two classes of sGC modulators exist, activators and stimulators, with differing sensitivity to oxidative stress. In this study, we investigated the therapeutic effects of the sGC stimulator BAY 41-2272 (Bay 41) and the sGC activator BAY 60-2770 (Bay 60) on endpoints of atherosclerosis and renal disease as well as inflammation and oxidative stress in diabetic Apolipoprotein E knockout (ApoE-/-) mice. We hypothesized that under oxidative conditions known to accompany diabetes, sGC activation might be more efficacious than sGC stimulation in limiting diabetic vascular complications. We demonstrate that Bay 60 not only significantly decreased nitrotyrosine staining (*P* < 0.01) and F4/80 positive cells by 75% (*P* < 0.05), but it also significantly reduced total plaque area (*P* < 0.05) and improved endothelial function (*P* < 0.01). Our data suggest an important anti-atherogenic role for Bay 60 accompanied by reduced oxidative stress and inflammation under diabetic settings. Treatment with the stimulator Bay 41, on the other hand, had minimal effects or caused no changes with respect to cardiovascular or renal pathology. In the kidneys, treatment with Bay 60 significantly lessened urinary albuminuria, mesangial expansion and nitrotyrosine staining under diabetic conditions. In summary, our head-to-head comparator is the first preclinical study to show that a sGC activator is more efficacious than a sGC stimulator for the treatment of diabetes-associated vascular and renal complications.

## 1. Introduction

Diabetes mellitus (DM) is a highly prevalent chronic metabolic disorder, characterized by elevated blood glucose, and considered a major health burden on western societies. Diabetes affects around 537 million people worldwide, and is predicted to reach 643 million by 2030 (1). Diabetic patients are highly susceptible to developing vascular complications, including atherosclerosis and chronic kidney disease leading to kidney failure. Often these occur as co-morbidities (2, 3) suggesting an underlying pathogenic etiology. These vascular complications are the major cause of morbidity and mortality in both Type 1 and Type 2 diabetic patients (4). There is currently no cure, despite standard treatments which include glucose and lipid lowering and blood pressure control.

A critical first step in the progression of vascular complications is the development of endothelial dysfunction, which is characterized by the reduced bioavailability of nitric oxide (NO) coupled with an elevation in oxidative stress (5). NO is a potent endogenous vasodilator that regulates vascular tone by increasing cGMP. NO is produced by endothelial nitric oxide synthase (eNOS) from L-arginine in the presence of co-factors HSP90, tetrahydrobiopterin (BH_4_) and the calcium-calmodulin complex. Once formed, NO diffuses to the vascular smooth muscle cells where it binds to soluble guanylate cyclase (sGC) which generates cyclic guanosine 3′,5′-monophosphate (cGMP). This NO-sGC-cGMP signalling pathway regulates numerous physiological downstream processes including vasodilation (6). NO-cGMP signalling is also anti-atherogenic via its ability to inhibit platelet aggregation, inflammatory cell adhesion and smooth muscle cell migration, all of which contribute to the pathogenesis of atherosclerosis (7, 8). Enhanced NO signalling is also associated with a higher glomerular filtration rate (GFR) as determined via measures of cystatin C and creatinine (9). Thus, increasing NO bioavailability is seen as an attractive therapeutic strategy to improve endothelial dysfunction, limit atherosclerosis and improve kidney function (10, 11).

Strategies to increase NO bioavailability particularly for diabetes-associated vascular complications, have faced major clinical limitations. For example, treatment with L-arginine has not shown improvements in endothelial function in diabetic patients (12), while nitrate administration has shown lack of sustainability due to the development of tolerance in humans (13). BH_4_ is easily oxidized and is temperature and light sensitive, limiting its use as a chronic drug treatment. In contrast, direct targeting of the sGC enzyme with small molecules has become the focus of a new treatment strategy to overcome the loss of NO bioavailability (14).

sGC is a heterodimeric enzyme, consisting of an alpha-subunit and a smaller heme-binding beta-subunit. Upon NO binding to the heme moiety, the enzyme becomes catalytically active. Under conditions of oxidative stress, ferrous (Fe^2+^) heme is oxidised to ferric (Fe^3+^) heme, leading to the loss of the heme group, which lessens or completely inhibits the response of sGC to NO by losing the NO-binding site (15). This is particularly prevalent under hyperglycemic conditions where the accumulation of oxidized heme-sGC results in a state of NO resistance, decreased NO-dependent cGMP accumulation and impaired vasodilation (16). Additionally, dyslipidemia, a common feature of diabetes leads to the dysregulation of sGC expression and activity, leading to vascular dysfunction and neointimal formation (16), both of which are key events in atherosclerotic progression.

Pharmacological compounds that directly target the sGC-cGMP pathway overcome limitations of NO tolerance. Two classes of compounds exist that increase the catalytic activity of the enzyme, namely sGC stimulators and sGC activators. Both sGC stimulators and sGC activators have a unique mode of action and can bind to sGC and trigger cGMP production independent of NO. In addition, sGC stimulators act by sensitizing sGC to low levels of NO by stabilizing the nitrosyl-heme complex. Thus, sGC stimulators also work synergistically with NO and upregulate sGC activity at reduced levels of NO (16, 17). In phase III clinical trials, Riociguat (BAY 63-2521), a sGC stimulator, has shown positive improvements in pulmonary arterial hypertension (18). In contrast to sGC stimulators, sGC activators modulate sGC when the enzyme is in an oxidized or a heme-free state (16, 17). Therefore, it is expected that sGC activators are advantageous under settings of oxidative stress such as diabetes, due to their ability to target heme-free sGC. However, in a comparative study by Costell et al. (19), both the sGC stimulator BAY 60-4552 and the sGC activator GSK2181236A demonstrated differential beneficial effects in spontaneous hypertensive stroke-prone rats, a model that is associated with high levels of oxidative stress.

Several studies have documented the protective effects of sGC modulation against atherosclerosis. For example, the sGC stimulator Riocuguat attenuated atherosclerosis in Western diet fed Apolipoprotein E knockout (ApoE-/-) mice (20). More recently it was shown that the sGC stimulator BAY-747 reduced atherosclerotic plaque formation in atherosclerosis-prone Ldlr−/− mice (21). In addition, sGC activation reduced cholesterol accumulation in macrophages by upregulating cholesterol efflux (22). Furthermore, both sGC stimulators and activators exhibit anti-fibrotic and anti-proliferative effects, which is of particular relevance to atherosclerotic plaque development (23–26). Several studies have also shown the renoprotective effects of sGC stimulators and activators in chronic kidney disease models (27, 28). However, little is known about the role of sGC modulation in diabetes-associated vascular complications.

Based on current knowledge, we aimed to show that sGC modulation will protect against diabetes-induced vascular dysfunction, atherosclerosis and nephropathy. Our preclinical studies directly compared the sGC stimulator, Bay 41-2272 (Bay 41), with the sGC activator, Bay 60-2770 (Bay 60) (17, 29). In particular, we hypothesized that under oxidative conditions known to accompany diabetes, sGC activation might be more efficacious than sGC stimulation in limiting diabetic vascular complications. Specifically, we aimed to investigate the effects of sGC stimulation or activation on cell types pertinent to the development of atherosclerosis (human aortic smooth muscle cells) under diabetic conditions. We also directly compared the effects of sGC stimulation or activation on vasodilation, atherosclerotic plaque development and renal function and injury in a mouse model of Type 1 diabetes.

## 2. Methods

### 2.1. Cell culture

Non-diabetic and diabetic human aortic smooth muscle cells (HAoSMC) from a single donor were purchased from Lonza Clonetics and cultured in smooth muscle cell media (SmBM-2, Lonza) in a humidified incubator at 37°C and 5% CO_2_. Cells were supplemented with 2% FBS at 37°C in 5% CO_2_.

#### 2.1.1. Proliferation assays of HAoSMCs treated with sGC activators and stimulators

To quantitate HAoSMC proliferation, diabetic and non-diabetic HAoSMC were seeded in 24-well tissue culture plates. After 24 h, cells were synchronised for an additional 24 h in serum-free media. Next, cells were stimulated with serum and TNF-α, with and without the addition of the sGC stimulator Bay 41 or the activator Bay 60, and analysed after a 48-hour period. Cells were counted using the TALI cytometer (Thermofischer). Additionally, a colorimetric water-soluble tetrazolium (WST)-1 assay (Roche Diagnostics) was used to measure cell proliferation.

#### 2.1.2. Gene expression of HAoSMCs treated with sGC activators and stimulators

Non-diabetic and diabetic HAoSMC were treated with TNF-α for 4 h in the presence or absence of the sGC stimulator Bay 41 or the activator Bay 60. Cells were harvested and RNA extraction, cDNA synthesis and qRT-PCR were performed as described previously (30). Gene expression analysis was performed to assess pro-inflammatory markers.

### 2.2. Animal groups and experimental design

To induce a model of Type 1 diabetes, eight-week old male ApoE^−/−^ mice were made diabetic with intraperitoneal injections of streptozotocin (STZ; 100 mg/kg on 2 consecutive days), dissolved in 25 mM citrate buffer at pH4.5. At 10 weeks of age, non-diabetic and diabetic (>25 mM blood glucose) mice were randomised to receive either the sGC stimulator Bay 41 (1 mg/kg and 10 mg/kg), the sGC activator Bay 60 (0.3 mg/kg and 3 mg/kg) or vehicle control (carboxy methylcellulose suspension) for a period of 10 or 20 weeks, via oral gavage, twice a day. For quantification of the exposure, blood (Lithium Heparin Plasma) was collected 1 h and 4 h after oral gavage and Bay 60 and Bay 41 were analyzed in plasma using an LC-system (Kinetex, 2.6 µm, C18 100 A LC Column 150 × 4.6 mm) coupled to a 4,500 Triple Quad Sciex mass analyzer (MS/MS), which was used in positive mode. The injection volume was 5 µl. An acetonitrile and ammonium acetate buffer (10 mM pH 6.8) gradient at a flow rate of 1 ml/min was used for mass separation. The generic internal standard was added to the samples before LCMS analysis and quality control samples were used to monitor the LCMS/MS quality. The 11-point calibration curve of Bay 60 and Bay 41 together with the internal standard was used for quantification.

After 10-weeks of treatment, mice were killed using lethabarb (100 mg/kg), and the aortas were dissected out for vascular function and gene expression analysis. After the 20 week end-point, mouse hearts and kidneys were weighed, and tibia length was determined. Blood was collected into heparinised tubes. 20 ul of blood was removed and white blood cells, neutrophils and monocytes were calculated using a Hemavet system (Drew Scientific). The remaining blood was centrifuged at 4,000 × g for 10 min at room temperature to separate plasma. Lipid composition was then analysed using a HPLC system (31). Aortae were dissected and assessed for the presence of atherosclerotic plaque by an *en face* method that included the pinning flat of the arch, thoracic and abdominal segments after Sudan IV staining to assess regional lesions (10). Additionally markers of renal damage were assessed by ELISA and immunohistochemistry.

### 2.3. Assessment of vascular function

Vascular function was assessed by myography as previously published by our group (32, 33). Briefly, the aorta was dissected out and cleaned of peripheral fat. Thereafter, two 4 mm segments of the thoracic aortae were mounted on two L-shaped metal prongs. Aortae were equilibrated for 30 min at a resting tension of 5 mN. All aortae were then exposed to high-potassium physiological salt solution (HPPSS) to determine viability. Next, cumulative concentration responses to acetylcholine (ACh; 1 nmol/L–100 µmol/L) were recorded in aortae preconstricted to −50% HPPSS with phenylephrine (PE). All vasorelaxation responses are presented as percentage relaxation of the preconstriction response. Additionally, a concentration-response curve to PE (1 nmol/L–100 µmol/L) was performed to assess vascular contractility. The variable slope sigmoidal concentration-response curves to all agonists for each mouse were calculated and plotted using GraphPad Prism (version 8.0).

### 2.4. Gene expression analysis

Total RNA was extracted from tissue after homogenization of snap frozen aortae and kidney. Gene expression of vascular cell adhesion molecule-1 (VCAM-1), intracellular adhesion molecule (ICAM-1), nuclear factor-*κ*B subunit p65 (p65 NF-*κ*B), monocyte chemoattractant protein-1 (MCP-1), interleukin-1β (IL-1β), and tumor necrosis factor-α (TNF-α) was analyzed by quantitative RT-PCR (qRT-PCR) as described previously (10).

### 2.5. Immunohistochemistry

Aortic and kidney nitrotyrosine as well as aortic F4/80 localization and expression were determined by immunohistochemistry. In brief, 4-µm paraffin sections mounted on Superfrost slides were dewaxed, and endogenous peroxidases were inactivated with 3% H_2_O_2_ in Tris-buffered saline. Antigen retrieval was performed for kidney sections. Thereafter, sections were incubated with a serum blocking agent and a biotin-avidin blocking kit (Vector Laboratories). Primary antibodies were added to sections and incubated overnight at 4°C. The next day, secondary antibody, biotinylated anti-rabbit Ig, or the biotinylated anti-rat secondary antibody was added for 30 min followed by horseradish peroxidase–conjugated streptavidin (1:500), incubated for 3 min in 3,3′-diaminobenzidine tetrahydrochloride, and counterstained with hematoxylin. All sections were examined under an Olympus BX-50 light microscope (Olympus Optical) and digital quantitation (Image-Pro Plus software version 6.0) and assessments were performed in a blinded manner. Nitrotyrosine staining was expressed as postively stained area over total area of the section while F4/80 positive cells were counted and averaged over sections.

### 2.6. Renal injury

Renal injury was assessed by measuring urinary albumin at midpoint (10 weeks of diabetes) and endpoint (20-weeks of diabetes) and PAS staining to quantify mesangial expansion. Urinary albumin was measured using a mouse albumin ELISA kit (Bethyl Laboratories) as per the manufacturer's instructions. PAS staining was performed and counterstained with hematoxylin. All sections were examined under an Olympus BX-50 light microscope (Olympus Optical) with digital quantitation (Image-Pro Plus software version 6.0) and assessments performed in a blinded manner.

### 2.7 Statistical analysis

Data are expressed as mean ± standard error of mean (SEM). A Shapiro-Wilk normality test was performed in GraphPad Prism to check for normality of data. If data is distributed normally, a one-way ANOVA with Tukey's multiple comparison *post hoc* tests was performed for comparisons between groups. If data was not normally distributed, a Kruskal-Wallis test was performed. All statistical analyses were performed using GraphPad Prism version 8.0. A *P* value < 0.05 was considered statistically significant.

## 3. Results

### 3.1. The sGC activator BAY60 and the stimulator BAY41 limit cell proliferation of human aortic smooth muscle cells in culture

**Figure 1:**
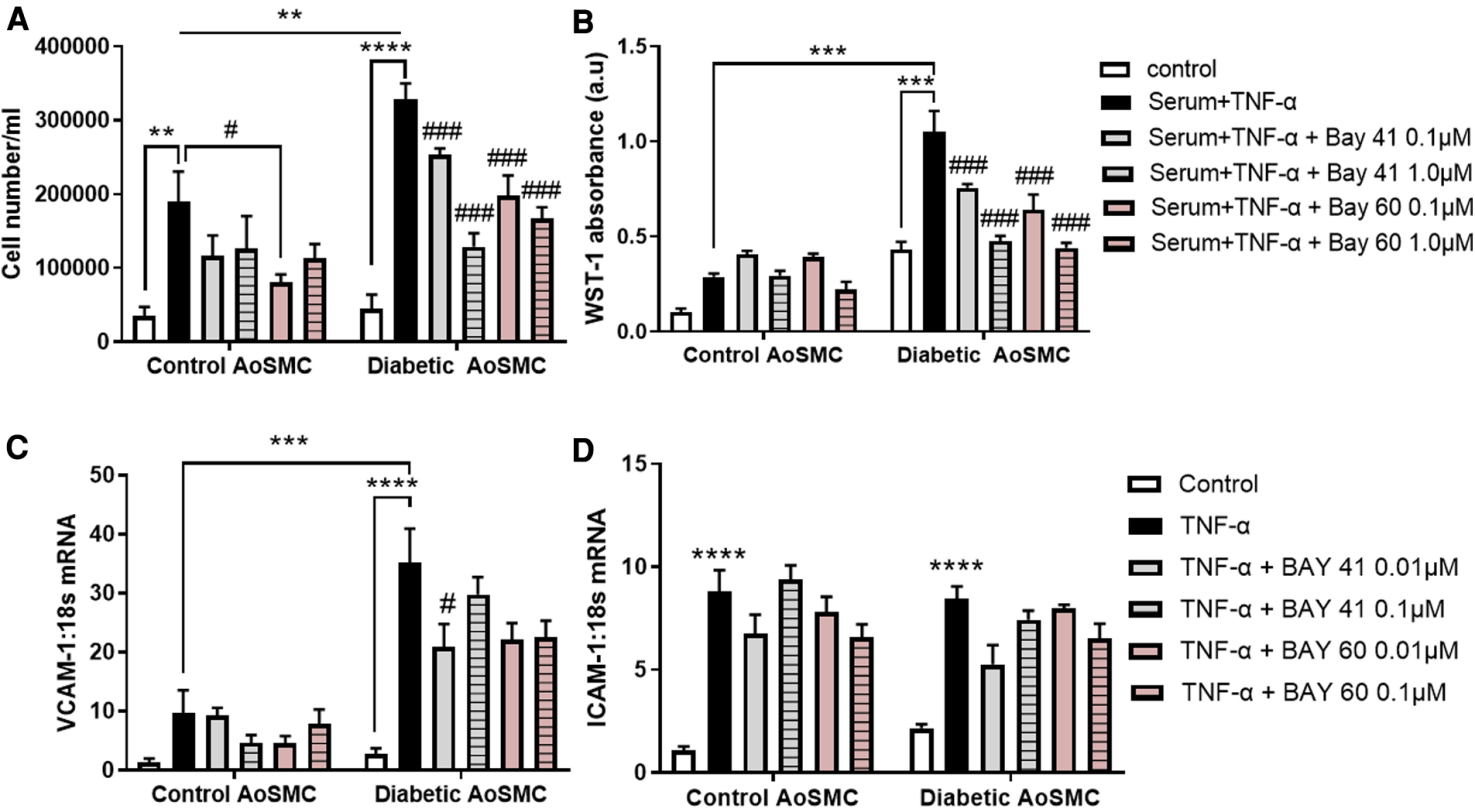
Treatment with Bay 41 and Bay 60 lessens cell proliferation in two independent assays. (**A**) Cell number and (**B**) a WST-1 proliferation assay of non-diabetic and diabetic HAoSMC treated with TNF-α and two doses of Bay 41 or Bay 60 as indicated. (**C,D**) Inflammatory gene expression analysis of (**C**) VCAM-1 and (**D**) ICAM-1 in non-diabetic and diabetic HAoSMC treated with TNF-α ± Bay 41 or Bay 60. Data is presented as mean ± SEM. *n* = 8/group with 2-3 independent experiments performed. #*P* < 0.05, **P < 0.01, ***P < 0.001 and ****P < 0.0001 as indicated. #*P* < 0.05, ##*P* < 0.01 and ###*P* < 0.001 vs. Diabetic HAoSMC + TNF-α.

Diabetic HAoSMC exhibited a significantly greater hyperproliferative profile in response to serum and TNF-α treatment as compared to non-diabetic HAoSMC (comparison of the black bars), as examined by both cell counting (Figure 1A, *P* < 0.01) and the WST-1 colorimetric assay (Figure 1B; *P* < 0.001).

In non-diabetic cells, Bay 60 caused a small yet significant reduction in cell number at 0.1 *μ*M (Figure 1A, *P *< 0.05). Importantly, treatment with Bay 41 and Bay 60 reduced proliferation in a dose-dependent manner in diabetic HAoSMC (Figures 1A,B; *P* < 0.001) when assessed by both cell counting and the WST-1 absorbance assay. Collectively, both Bay 41 and Bay 60 inhibited cellular proliferation of diabetic human aortic smooth muscle cells in a dose-dependent manner with the higher concentration of 1*μ*M being more effective.

### 3.2. sGC activator Bay 60 and stimulator Bay 41 lessen inflammation

Diabetic HAoSMC showed increased expression of the pro-inflammatory marker, VCAM-1 (Figure 1C; *P* < 0.001) compared with non-diabetic HAoSMC. Bay 41 (0.01 μM) significantly lessened VCAM-1 gene expression in diabetic HAoSMC (Figure 1C). There was a tendency to lessen VCAM-1 gene expression by Bay 60 at both concentrations in treated diabetic HAoSMCs compared with untreated diabetic HAoSMCs although significance was not reached (*P* value = 0.1).

ICAM-1 was significantly increased in both non-diabetic and diabetic HAoSMC, however Bay 41 and Bay 60 treatment had no significant effect on ICAM-1 expression (Figure 1D). A trend towards decrease expression after BAY41 (0.01 μM) treatment in both control and diabetic cells (*P* = 0.06) was observed. A dose-dependent trend was also observed after Bay 60 treatment in diabetic HAoSMCs.

Taken together, these *in vitro* experiments in diabetic HAoSMCs suggest that soluble guanylate cyclase activators and stimulators limit HAoSMC growth and the diabetes-mediated pro-inflammatory phenotype.

### 3.3 Plasma exposure of Bay 60 and Bay 41 after oral dosing in ApoE -/-mice

After oral dosing, plasma exposure of the sGC activator Bay 60 and the sGC stimulator BAY41 was determined 1, 2 and 4 h post treatment in ApoE -/- mice. Overall, plasma concentrations of Bay 41 were dose linear between 1 and 10 mg/kg oral doses over time (Supplementary Figure S1A). Plasma concentrations of Bay 60 showed no dose-linear increase in exposure after 3 mg/kg compared to 0.3 mg/kg (see Supplementary Figure S1A).

### 3.4. sGC activation improves vascular function and lessens oxidative stress

**Figure 2:**
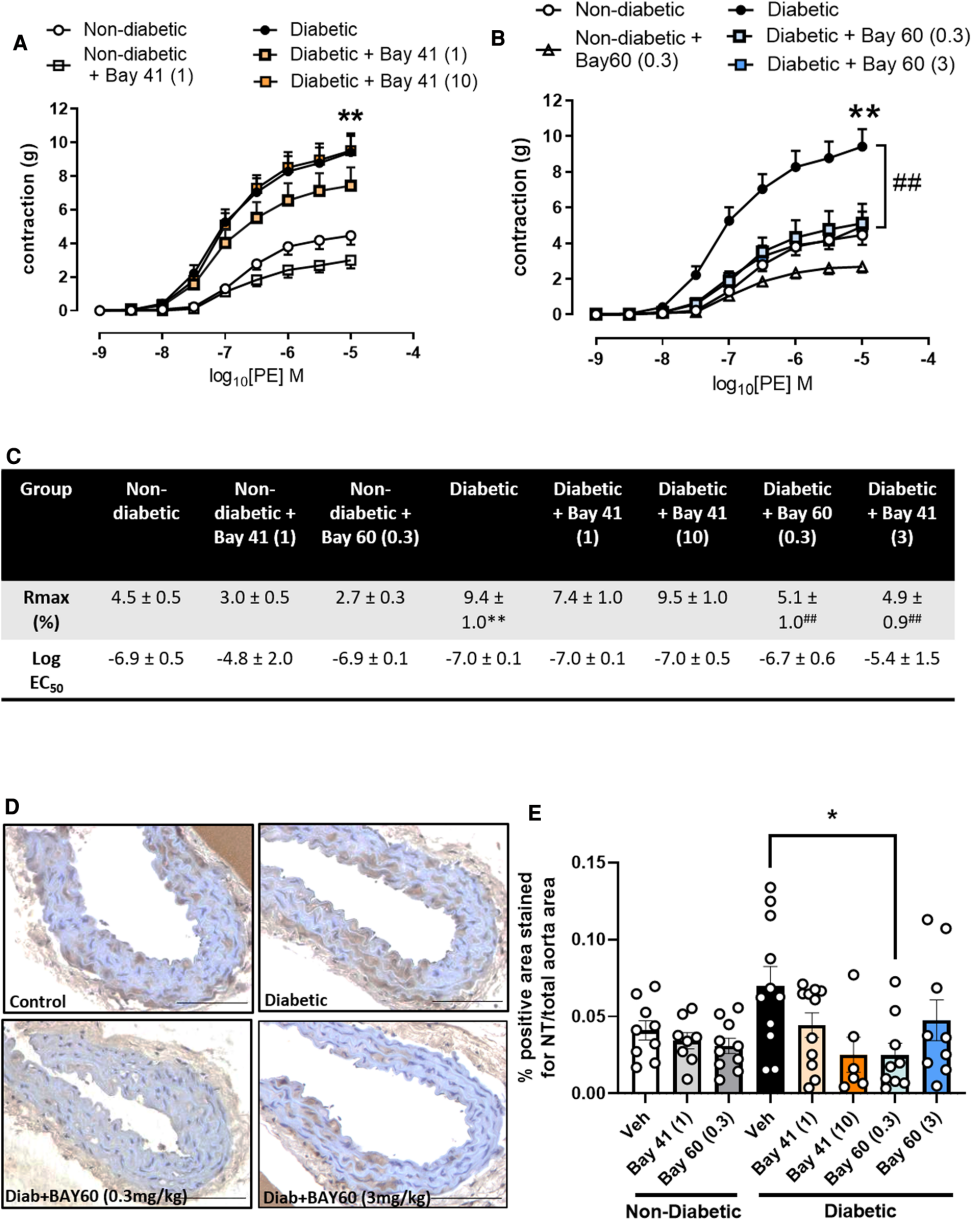
(**A,B**) vascular contraction in response to increasing doses of phenylephrine (PE) in aortic vessels treated with (**A**) Bay 41 and (**B**) Bay 60. (**C**) Rmax and Log EC50 values for PE concentration-response curves were calculated and used for statistics. (**D**) Representative images of aortic sections stained with nitrotyrosine and (**E**) quantification of nitrotyrosine stained positive area. Data is presented as mean ± SEM, with individual values plotted. *P < 0.05 as indicated, **P < 0.01 Diabetic vs. Non diabetic; ##*P* < 0.01 Diabetic + Bay 60 (0.3) vs. Diabetic. *n* = 6-13 per group. Dose (mg/kg) of Bay 41 and Bay 60 indicated in brackets.

After 10-weeks of treatment with either Bay 41 or Bay 60, non-diabetic and diabetic vessels were assessed for improvements in vascular function. Diabetic vessels (black circles) exhibited a significantly greater contraction in response to increasing concentrations of phenylephrine (PE) compared with non-diabetic vessels (open circles) (Figures 2A,C, *P* < 0.01), suggesting significant diabetes-induced vascular dysfunction. Treatment with Bay41 did not significantly affect PE contractility at both doses although a small reduction was observed at 10 mg/kg (Figures 2A,C). Treatment with Bay 60 reduced PE contractility both at 0.3 mg/kg and 3 mg/kg (Figures 2B,C, *P* < 0.01), suggesting that both doses, which showed similar plasma exposure of the sGC activator, protected against diabetes-mediated vascular dysfunction. In non-diabetic vessels, both Bay 41 and Bay 60 showed a small but not significant reduction in PE contractility (Figures 2A,B). Interestingly, there was no effect of sGC treatment on the responses to acetylcholine (Ach) in diabetic or non-diabetic vessels (Supplementary Figure S3), suggesting that improvement in vascular function by the sGC activator is driven mainly via positive effects on vascular smooth muscle cells.

Vascular oxidative stress was evaluated via immunohistochemical staining for nitrotyrosine, a marker of peroxynitrite induced oxidative damage (Figures 2D,E). Nitrotyrosine staining showed a trend towards increased expression in diabetic aortae compared with non-diabetic aortae (*P* = 0.1). Treatment with Bay 60 (0.3 mg/kg) significantly attenuated the extent of nitrotyrosine staining (*P* < 0.05) (Figures 2D,E) while a trend towards decreased expression was noted after Bay 41 (10 mg/kg) treatment, suggesting that sGC activation and stimulation lessens oxidative stress.

### 3.5 End-point metabolic parameters

End-point parameters were assessed after 20-weeks of Bay 41 and Bay 60 treatment. There was no significant difference in body weight between non-diabetic and diabetic mice (Table 1). Non-diabetic mice (vehicle treated) had a mean body weight of 25.1 g ± 0.6 and diabetic mice had a mean body weight of 26.4 g ± 0.7. Treatment with the sGC activators and stimulators at either dose had no effect on the body weights of the mice.

**Table 1:** Blood pressure, blood glucose, body weight, height weight and kidney weight at study end point (20 weeks).

Parameter	Non-diabetic	Non-diabetic + Bay41	Non-diabetic + Bay60	Diabetic	Diabetic + Bay41	Diabetic + Bay41	Diabetic + Bay60	Diabetic + Bay41
		1 mg/kg	0.3 mg/kg		1 mg/kg	10 mg/kg	0.3 mg/kg	3 mg/kg
Blood pressure (mmHg)	99.8 ± 4.2	94.0 ± 7.2	116.1 ± 9.0	101.0 ± 3.9	110.4 ± 4.2	96.7 ± 8.8	109.3 ± 5.0	106.5 ± 5.0
Blood glucose (mmol/L)	9.8 ± 0.4	10.9 ± 0.8	9.6 ± 0.5	26.2 ± 2.4*	26.2 ± 2.4*	21.7 ± 3.7*	20.0 ± 3.0*	24.8 ± 3.0*
Body weight (g)	25.1 ± 0.1	28.8 ± 0.1	28.3 ± 0.1	26.4 ± 0.6	26.7 ± 0.6	28.0 ± 0.4	28.6 ± 0.7	27.5 ± 0.7
Heart weight (g)	0.1790 ± 0.0268	0.1414 ± 0.0054	0.1483 ± 0.0071	0.1416 ± 0.0071	0.1276 ± 0.0047	0.1282 ± 0.0044	0.1313 ± 0.0043	0.1261 ± 0.0038
Heart weight/Tibia length (g/mm)	0.0080 ± 0.0004	0.0089 ± 0.0009	0.0084 ± 0.0004	0.0080 ± 0.0004	0.0073 ± 0.0003	0.0072 ± 0.0003	0.0073 ± 0.0002	0.0077 ± 0.0007
Right kidney weight (g)	0.1814 ± 0.0055	0.1735 ± 0.0054	0.1782 ± 0.0074	0.1969 ± 0.0116	0.1954 ± 0.0082	0.1856 ± 0.0119	0.1763 ± 0.0049	0.1886 ± 0.0053
Left kidney weight (g)	0.1739 ± 0.0049	0.1658 ± 0.0061	0.1723 ± 0.0082	0.1818 ± 0.0060	0.1959 ± 0.0103	0.1810 ± 0.0094	0.1701 ± 0.0057	0.1851 ± 0.0055

Data is expressed as mean ± SEM.

* *p* < 0.001 vs. Non-diabetic.

Blood pressure was measured before termination using a tail cuff method (Table 1). There was no observed change in blood pressure between non-diabetic and diabetic mice. Furthermore, treatment with the sGC stimulator Bay 41 or the activator Bay 60 had no effect on blood pressure as observed by tail cuff measurements.

The ratio of heart weight to tibia length remained unaltered with the induction of diabetes and treatment with sGC modulators (Table 1), suggesting that there was no hypertrophy of the heart in this study, and that treatment with the stimulators and activators had no effect on heart size. There was a slight trend towards increased right kidney weight in the diabetic groups but this was unaltered with sGC modulation (Table 1).

Diabetic mice regardless of treatment exhibited elevated blood glucose levels compared with non-diabetic mice (Table 1; *P* < 0.001). Additionally, diabetic mice (black bars) exhibited elevated cholesterol and LDL/HDL ratios compared with non-diabetic mice (Supplementary Figures S2A–E). Importantly, treatment with either Bay 41 or Bay 60 did not affect lipid parameters (Supplementary Figure S2C).

### 3.6. Bay 60 but not Bay 41 significantly lessens atherosclerosis

**Figure 3:**
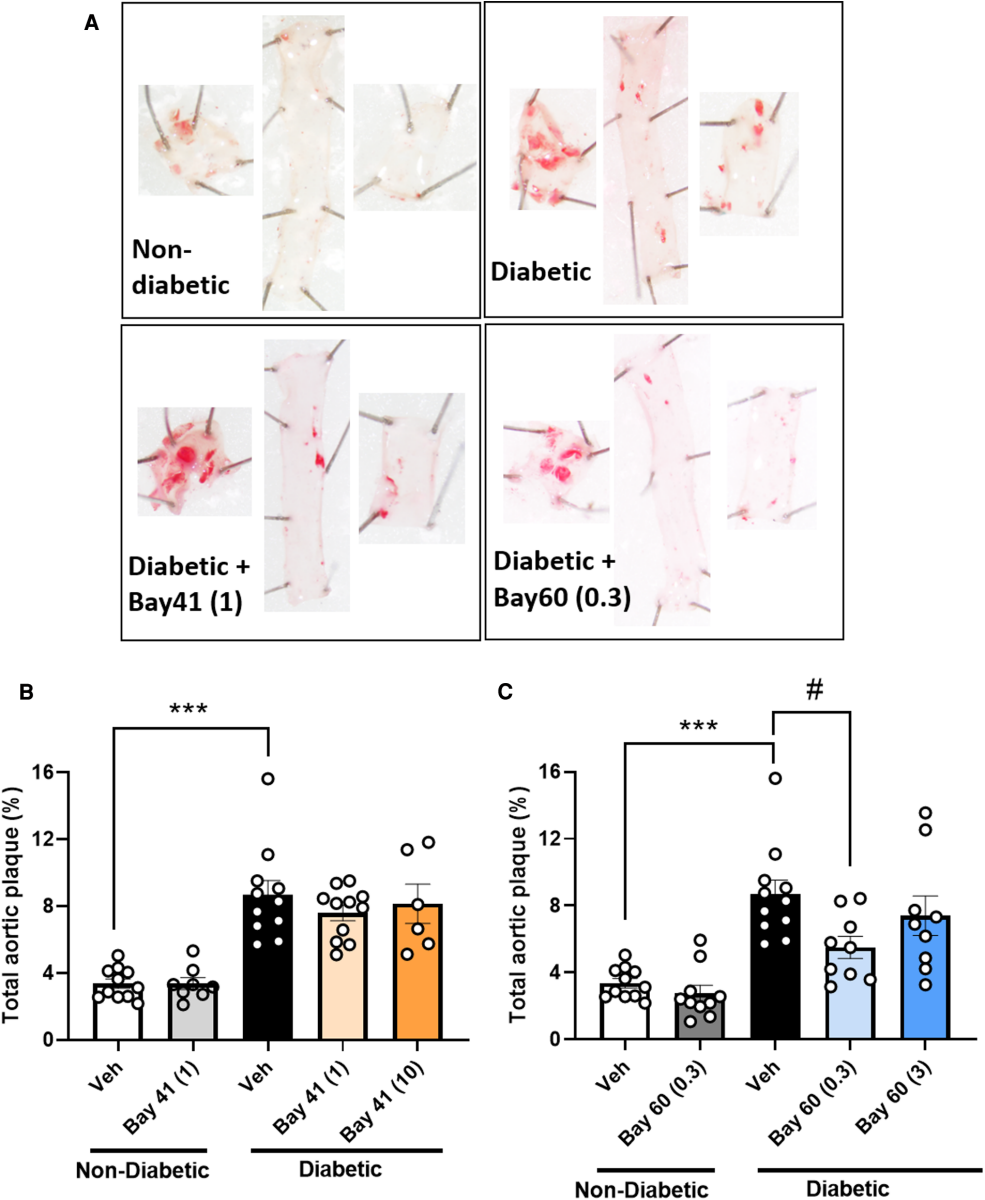
Sudan IV-stained aortas and atherosclerotic plaque quantification from mice treated with Bay 41 and Bay 60. (**A**) Representative images and percentage total plaque is shown for (**B**) Bay 41 treated and (**C**) Bay 60 treated ApoE-/- mice. Data is presented as mean ± SEM, with individual values plotted. ***P < 0.001 and #*P* < 0.05 as indicated. *n* = 6-12 aortas analysed per group. Dose (mg/kg) of Bay 41 and Bay 60 indicated in brackets.

Diabetic aortas displayed an approximately 2.5-fold increase in total aortic plaque compared with non-diabetic aortas (Figure 3; *P* < 0.001). Treatment with Bay 41 did not affect the percentage total plaque (Figure 3B), whereas treatment with Bay 60 significantly reduced atherosclerotic lesions in diabetic vessels at 0.3 mg/kg (Figure 3C, *P* < 0.05).

### 3.7. Diabetes-driven myelopoesis is lessened by sGC treatment

**Figure 4:**
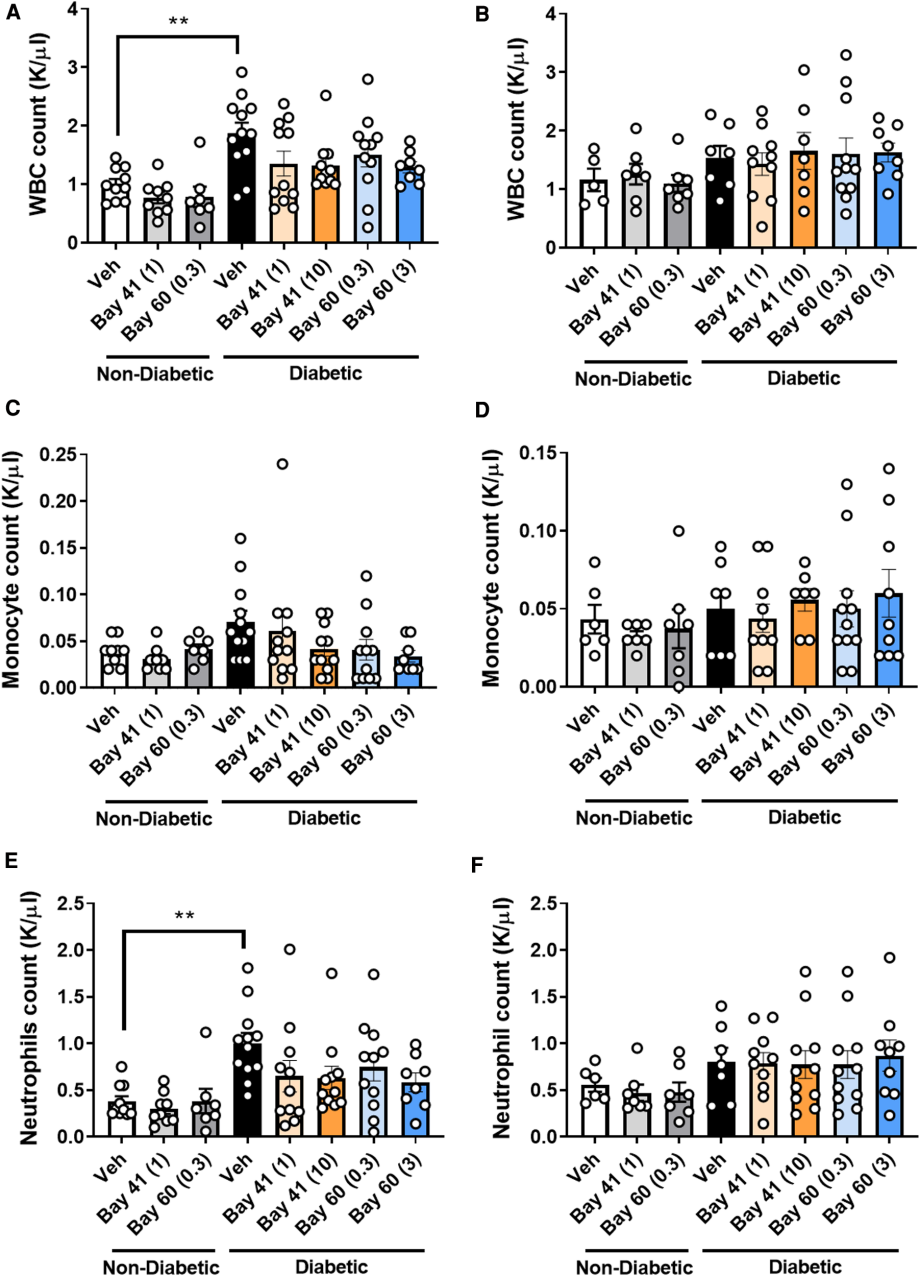
White blood cell (WBC), monocytes and neutrophils counts at 10 week (**A**, **C** and **E** respectively) and 20 week time points (**B**, **D** and **F** respectively). Data is presented as mean ± SEM, with individual values plotted. **P < 0.01 as indicated. *n* = 6–13 per group. Dose (mg/kg) of Bay 41 and Bay 60 indicated in brackets.

Blood from diabetic mice (Figures 4A,B; black bars) exhibited significantly elevated total white blood cell (WBC) count at the 10-week time point compared with non-diabetic mice, which was not observed at the 20-week time point (Figure 4B). More in-depth analysis of the subtypes of WBCs showed significantly elevated neutrophils in the blood of diabetic mice after 10 weeks of diabetes (Figure 4E), whilst other WBCs such as monocytes were unaffected by diabetes (Figure 4C). This suggests that diabetes drives an early robust myelopoesis, mainly driven by elevated neutrophils in these mice, in line with published data (34). Treatment with the sGC compounds Bay 41 or Bay 60 tended to prevent the diabetes-driven myelopoesis at the earlier time point (10-weeks after commencement of treatment), although this did not fall within statistical significance (Figures 4A,C,E; *P* = 0.2 between diabetic vehicle vs. diabetic + Bay41 (10 mg/kg) and diabetic + Bay60 (3 mg/kg)). Interestingly, neither Bay 41 nor Bay 60 had any impact on WBC count at the 20-week time point (Figure 4B), suggesting that these compounds limit early myelopoesis, and in particular neutrophil accumulation in the blood. This is in line with the known function of neutrophils as early responders that migrate towards sites of inflammation where their secretion products activate monocytes that then enter atherosclerotic lesions or release pro-inflammatory mediators (35).

### 3.8. sGC activation with BAY60 lessens vascular inflammation

**Figure 5:**
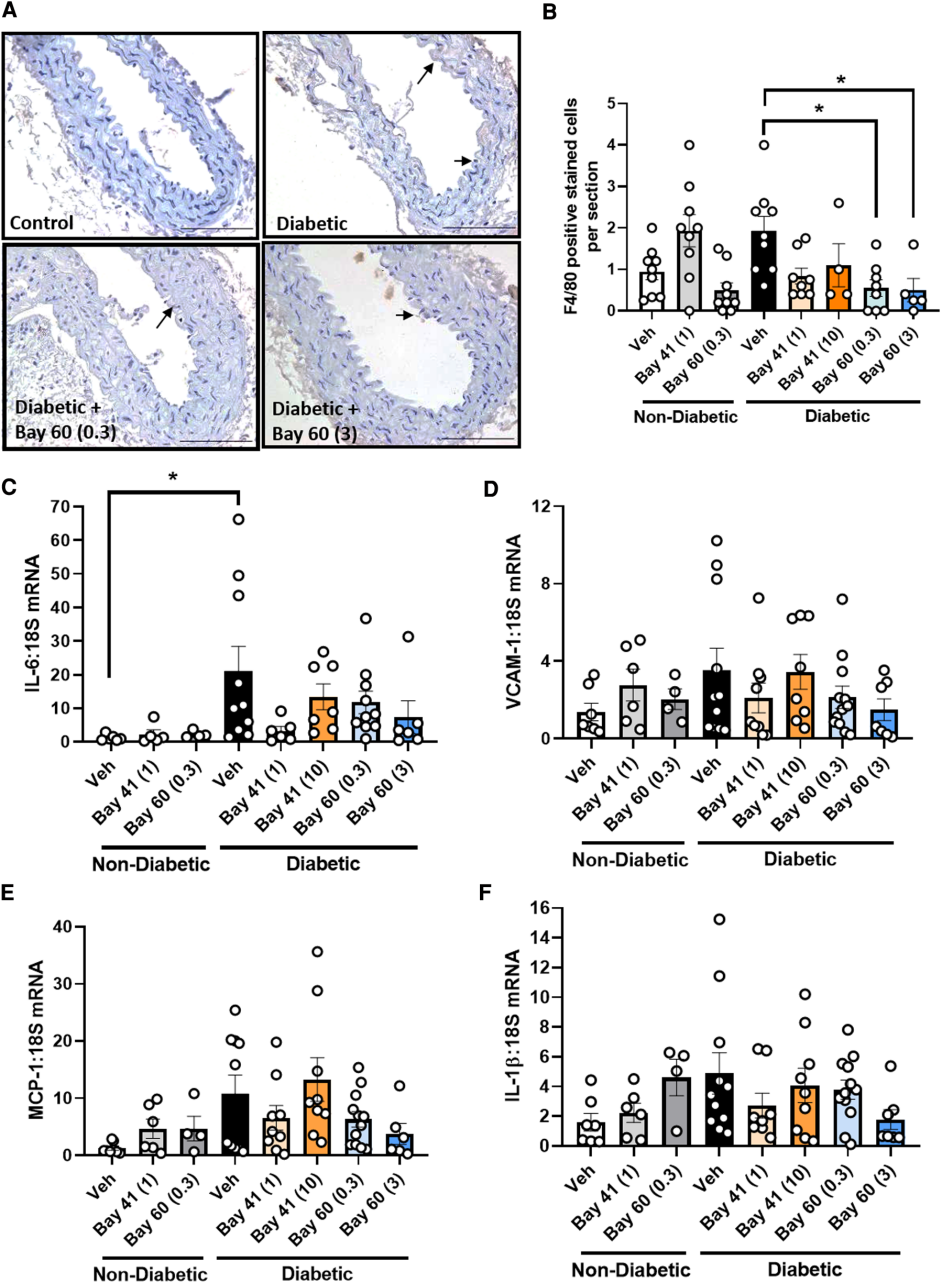
(**A**) Representative images of aortic sections stained with macrophage maker F4/80 and (**B**) quantification of F4/80 positive cells by averaging cell number across 10 sections per aorta. Gene expression of aortic inflammatory markers: (**C**) IL-6, (**D**) VCAM-1, (**E**) MCP-1 and (**F**) IL-1β was assessed by qRT-PCR. Data is presented as mean ± SEM, with individual values plotted. *P < 0.05 as indicated. *n* = 6–13 per group. Dose (mg/kg) of Bay 41 and Bay 60 indicated in brackets.

Macrophage infiltration, a marker of inflammation, was quantified in aortic sections using a murine-specific antibody against the macrophage marker, F4/80. Diabetic aortae exhibited a trend towards an increase in positively stained cells compared with non-diabetic aortae (Figures 5A,B, *P* = 0.17). Treatment with Bay 60 at 0.3 mg/kg and 3 mg/kg significantly reduced the number of macrophages in the diabetic aorta by −75% (Figures 5A,B, *P* < 0.05). A trend towards a decrease in macrophage numbers was observed after 1 mg/kg of Bay 41 treatment, however, this failed to reach significance (Figure 5B, *P* = 0.1).

Next we assessed a range of inflammatory genes (IL-6, VCAM-1, MCP-1 and IL-1β) by qRT-PCR (Figures 5C–F). The expression of IL-6 was significantly elevated in diabetic aortae (black bars) and VCAM-1, MCP-1 and IL-1β trended upward in gene expression in diabetic aortae, whilst treatment with Bay 60 at both doses (0.3 and 3 mg/kg) caused a trend toward reduced expression of these inflammatory genes (Figures 5C–F). There was also a trend towards decreased gene expression with Bay 41 at the lower treatment dose of 1 mg/kg although this was not apparent at the higher dose of 10 mg/kg (Figures 5C–F, *P* = 0.13 between diabetic vehicle vs. diabetic + Bay 41 (1 mg/kg) and diabetic + Bay 60 (3 mg/kg))**.**

### 3.9. Renal function (albuminuria) is improved after sGC activator and stimulator treatment

**Figure 6:**
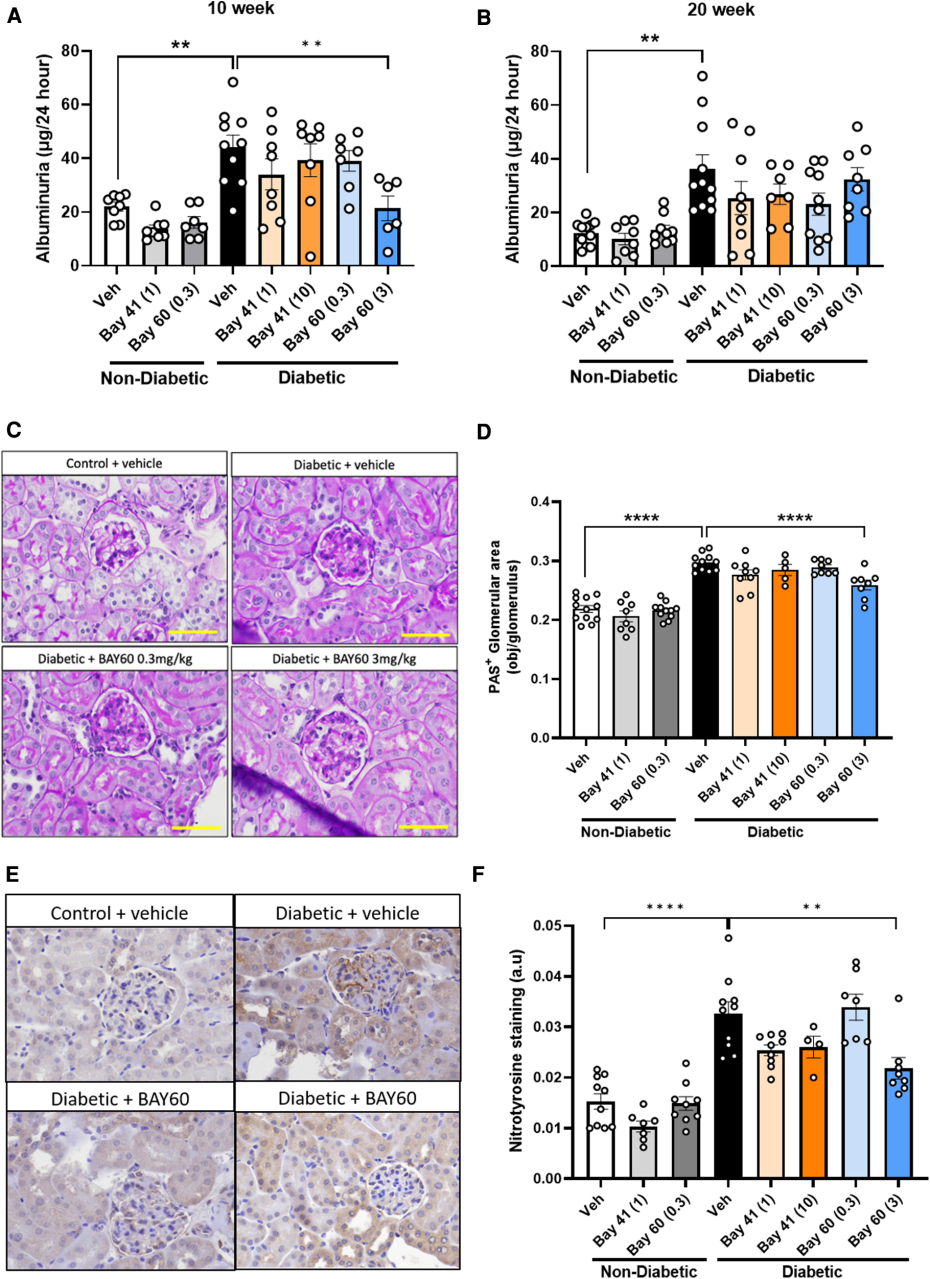
Urinary albuminuria was quantified using a commercially available ELISA kit. Total urine production over 24 h was used to determine final albuminuria levels (expressed as μg per 24 h) at (**A**) 10 weeks and (**B**) 20 weeks. (**C**) Representative images and (**D**) Quantification of kidney sections stained with Periodic acid-Schiff (PAS) in diabetic and non-diabetic mice (Magnification = 400x, scale bar = 50 μm). Results are shown as percentage-positive stained glomerular area and 20 glomeruli per kidney were analyzed. (**E**) Representative immunohistochemical images and (**F**) Quantification of glomeruli stained for nitrotyrosine in diabetic and non-diabetic ApoE-/- mice (magnification = 400x, scale bar = 50 μm). Results shown as percentage-positive brown staining per glomerulus area. 20 glomeruli per kidney section were analysed. Data is presented as mean ± SEM, with individual values plotted. **p < 0.01 and ****p < 0.0001 as indicated. *n* = 5–11per group. Dose (mg/kg) of Bay 41 and Bay 60 indicated in brackets.

Elevated proteinuria is a functional marker for the development of diabetic nephropathy. Thus, urine albuminuria levels were measured using ELISA. A highly significant increase in albuminuria (−2-fold) was detected in urine from diabetic mice compared with urine from non-diabetic mice at both the 10 and 20-week time points (Figure 6).

Compared to untreated diabetic mice, Bay 41 and Bay 60 showed a trend towards reduced albuminuria levels for all treated groups at both the 10- and 20-week time point. This downward trend was significant after 10 weeks of treatment with Bay 60 at 3 mg/kg (*P *< 0.01). Our data suggest that the sGC activator Bay 60, at a dose of 3 mg/kg, improves renal function in diabetic mice after 10 weeks of treatment.

### 3.10. Renal structural injury is reduced by BAY60 treatment

Histological examination of periodic acid-Schiff (PAS) stained kidney sections showed extensive glomerular pathology and renal injury after 20 weeks of diabetes. Diabetic kidneys showed basement membrane thickening and glomerular hypertrophy due to mesangial expansion and increased extracellular matrix deposition (ECM) compared with non-diabetic kidney sections. This is visualized as dark-magenta stained regions in the glomeruli of the diabetic kidneys (Figure 6C). Histological analysis showed a statistically significant increase in PAS staining in the untreated diabetic group (black bar) compared with untreated non-diabetic and treated non-diabetic controls (Figures 6C,D; *P* < 0.0001). Treatment with Bay 60 at 3 mg/kg caused a statistically significant decrease in glomerular ECM deposition in the diabetic kidney (Figures 6C,D; *P* < 0.001), suggesting that Bay 60 at 3 mg/kg protects against structural injury in diabetic mice.

### 3.11. Renal oxidative stress

Diabetes-mediated oxidative stress was detected using immunohistochemical staining for nitrotyrosine. Vehicle-treated diabetic mice showed a significant increase in nitrotyrosine staining compared to all control non-diabetic groups (Figures 6E,F; *P* < 0.0001).

Treatment with Bay 41 at both doses did not significantly alter nitrotyrosine levels despite a strong decreasing trend. Treatment with Bay 60 (3 mg/kg) significantly attenuated nitrotyrosine staining compared to vehicle-treated diabetic mice (Figures 6E,F; *P* < 0.001), suggesting that Bay 60, at 3 mg/kg, protects against diabetes-driven oxidative injury.

### 3.12. cGMP levels as an indication of sGC activity

cGMP levels, a measure of sGC activity, were analysed in the plasma of diabetic and non-diabetic mice after 20 weeks of treatment with BAY compounds. Compared with non-diabetic untreated controls, cGMP levels were unchanged by 20 weeks of diabetes (Supplementary Figure S1B). Similarly, treatment with Bay 41 and Bay 60 (0.3 mg/kg) did not significantly alter cGMP levels. sGC activation with Bay 60 at a dose of 3 mg/kg showed a tendency towards an increase in cGMP levels in the plasma of diabetic mice when compared to untreated diabetic mice, however this did not reach significance. Additionally, sGC activation with Bay 60 at the 3 mg/kg dose was significantly higher than untreated non-diabetic mice (Supplementary Figure S1B; *P* < 0.05).

## 4. Discussion

This study aimed to establish whether sGC modulation, using the sGC stimulator Bay 41 and the sGC activator Bay 60, has the potential to protect against diabetes-associated atherosclerosis and renal damage. Using a preventative model where drug treatment commenced at the time of diabetes initiation, we demonstrated that the sGC activator, Bay 60 (0.3 mg/kg), was more efficacious than the stimulator, Bay 41, in reducing atherosclerotic plaque. This was accompanied by significant reductions in inflammation (F4/80 macrophages) and oxidative stress (nitrotyrosine) within the plaque and vasculature of diabetic mice. This atheroprotective effect was complemented by improvements in vascular function as demonstrated by reduced contractility in response to the vasoconstrictor, phenylephrine, as well as a reduction in smooth muscle cell proliferation under diabetic conditions. Additionally, Bay 60 demonstrated strong tendencies to reduce pro-inflammatory cytokines IL-6 and IL-1β, as well as the macrophage recruiting adhesion molecules, ICAM-1 and VCAM-1, under diabetic conditions. On the contrary, even though Bay 41 demonstrated modest decreases in vascular inflammation markers, it did not have an impact on vascular function and the atheroprotective effects were less prominent. The lack of impact of Bay 41 on vascular contraction could explain the differences in atheroprotective effects observed between the sGC stimulator and activator. A more patent vessel is correlated with enhanced and smoother blood flow, reduced endothelial damage due to reduced shear stress, and improved nutrient supply to the underlying vasculature. These factors, as a consequence of greater vessel relaxation by Bay 60, may have contributed to the decrease in plaque size observed. Additionally, it is of interest to note that the higher dose of the activator Bay 60 (3 mg/kg) did not reduce plaque size. Although the precise mechanisms have not been evaluated in this study, our data would suggest that a therapeutic window exists wherein Bay 60 is optimal.

With respect to renal pathology, our study showed significantly increased extracellular matrix protein accumulation and fibrosis under hyperglycaemic conditions. Fibrosis involves vascular smooth muscle cell proliferation, extracellular matrix accumulation and inhibition of matrix degradation (17). In our study, fibrosis in the kidney was lessened by Bay 60 as demonstrated by significant reductions in PAS staining. Additionally, albuminuria, an indicator of renal function, was significantly improved with Bay 60 treatment. These improvements were accompanied by a reduction in oxidative stress as demonstrated by reduced nitrotyrosine staining in the diabetic kidney upon treatment with the sGC activator Bay 60. Interestingly, Bay 60 protected against diabetes-mediated atherosclerosis at the lower dose of 0.3 mg/kg but prevented kidney injury at 3 mg/kg. Thus, we speculate that the metabolism of Bay 60 may be different between these tissues such that a higher dose is required in the kidney to elicit its protective effect.

This anti-fibrotic effect of Bay 60 ties in with the known anti-fibrotic effect of other sGC modulators (36, 37). Mechanistically, sGC modulation and associated cGMP release have been shown to block non-canonical TGF*β* signaling and downstream experimental fibrosis (33) as well as attenuating cell proliferation in a variety of cultured cell lines including primary arterial smooth muscle cells (26). This additional anti-fibrotic function of activated sGC and its effector molecule cGMP is expected to limit the progression of atherosclerosis and nephropathy in diabetic patients and would be a highly desirable outcome of early therapy. In support of this, sGC stimulation by BAY 41-2272 has been shown to decrease ventricular interstitial fibrosis and collagen accumulation in a hypertensive rat model (23), while the sGC activator, ataciguat, attenuated extracellular matrix accumulation in the non-infarcted ventricle of rats with heart failure and myocardial infarction (24). Additionally, BAY 41-2272 significantly reduced pulmonary arterial smooth muscle proliferation and migration *in vitro* and reduced neotimal growth post- vascular balloon injury *in vivo* (25, 26).

In another study that compared the efficacy of activators and stimulators under non-diabetic settings, low dose non-hypotensive treatment with the sGC stimulator, BAY 60-4552, improved renal function and survival, whilst the sGC activator, GSK2181236A, reduced cardiac hypertrophy. At high doses, both compounds attenuated cardiac hypertrophy but only the sGC stimulator further improved renal function and attenuated mean arterial pressure. These results suggested that activators and stimulators act preferentially in different tissues. Furthermore, neither sGC stimulation nor activation improved endothelium-dependent vasodilatory responses, suggesting that improvements due to sGC activity act downstream within the smooth muscle cells (19). Our study is in agreement with this finding since we failed to show improvements in vascular function in response to acetylcholine. Collectively, these results suggest that clinical development may need to take into account tissue-specific changes in the oxidation of sGC that are observed in different disease models.

Additionally, recent evidence has highlighted a role for sGC in regulating cholesterol metabolism in macrophages, another prominent cell type involved in the inflammatory component of atherosclerosis development. In atheroprone animal models, sGC inhibition significantly promoted ox-LDL induced cholesterol accumulation in macrophages (22), suggesting that sGC stimulation might prevent macrophage lipid accumulation and cytokine production. Furthermore, evidence from renal biopsies has shown that macrophage accumulation in diabetic kidneys predicts declining renal function (38).

Recent evidence also suggests that the loss of sGC function in platelets contributes to atherosclerotic plaque formation which could be reversed by chronic treatment with a sGC stimulator (21), suggesting that the effects we observed might also be mediated via rescued platelet function. Importantly, our data is in agreement with the anti-atherogenic potential of sGC agonists and it is clear that the sGC enzyme plays a critical role in regulating several cell types and processes involved in atherogenesis and nephropathy, including vascular tone, fibrosis and inflammation, and that targeting this enzyme could prove beneficial for the overall health of the vascular endothelium and prevent renal disease progression.

In conclusion, our head-to-head comparator is the first preclinical study that has shown that the sGC activator Bay 60 is superior to Bay 41 and that Bay 60 has potential therapeutic promise in the treatment of diabetes-associated vascular complications. It is well known that sGC stimulators and activators act via different mechanisms. sGC stimulators act by sensitizing sGC to low levels of NO by stabilizing the nitrosyl-heme complex, whilst sGC activators activate sGC when the enzyme is in an oxidized or in a heme-free state. This advantage of sGC activators is particularly relevant in diabetic patients where endogenous intravascular NO synthesis is markedly decreased and oxidative stress is increased (5, 39). Thus, as highlighted by our study, the sGC activator Bay 60 is more likely to be advantageous in settings of oxidative stress, such as those observed in cardiovascular diseases and diabetes, as a consequence of targeting heme-free sGC. Indeed, our collective data suggest that Bay 60 is the better of the two sGC enhancers at limiting vascular complications under diabetic conditions.

## Data Availability

The original contributions presented in the study are included in the article/**Supplementary Material**, further inquiries can be directed to the corresponding author.

## Supplementary material

Supplementary Figure S1 (**A**) Plasma exposure to the sGC activator BAY60 and the sGC stimulator BAY41 in ApoE-/- mice after oral dosing over a 4hr period. (**B**) cGMP levels, a measure of sGC activity analysed by ELISA in the plasma of diabetic and non-diabetic mice. Data is presented as mean ± SEM with individual values plotted. **P *< 0.05 as indicated. Dose (mg/kg) of Bay 41 and Bay 60 indicated in brackets.

Supplementary Figure S2 Lipid parameters at study end-point (20 weeks) assessed via HPLC. Data is presented as mean ± SEM, with individual values plotted. **P *< 0.05 and ****P *< 0.001 as indicated. *n *= 6-13 per group. Dose (mg/kg) of Bay41 and Bay60 indicated in brackets.

Supplementary Figure S3 (**A,B**) Vascular relaxation in response to increasing doses of Acetylcholine (Ach) in aortic vessels treated with (**A**) Bay 41 and (**B**) Bay 60. Data is presented as mean ± SEM. Dose (mg/kg) of Bay 41 and Bay 60 indicated in brackets.
