# Supplementary figures and images for: Ameliorating diabetes-associated atherosclerosis and diabetic nephropathy through modulation of soluble guanylate cyclase
Source: Front Cardiovasc Med. 2023 Jul 12;10:1220095. doi: 10.3389/fcvm.2023.1220095 (PMC10368983; doi:10.3389/fcvm.2023.1220095)

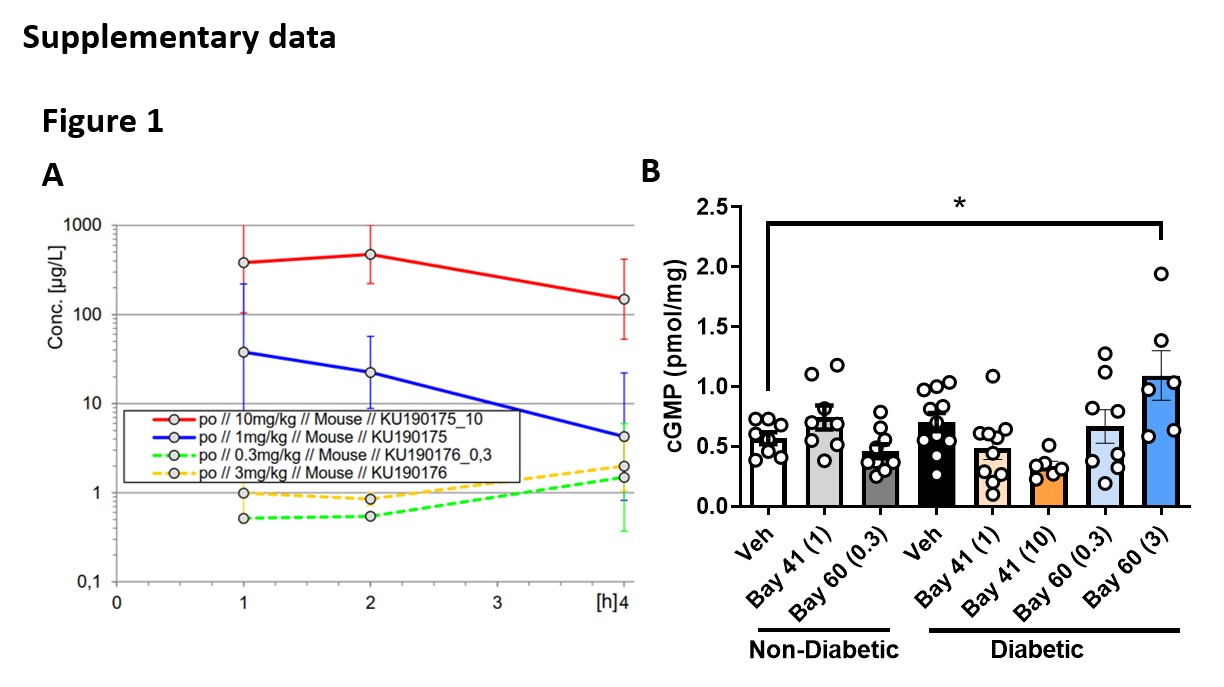

Supplement: Supplementary Figure S1 — (A) Plasma exposure to the sGC activator BAY60 and the sGC stimulator BAY41 in ApoE-/- mice after oral dosing over a 4hr period. (B) cGMP levels, a measure of sGC activity analysed by ELISA in the plasma of diabetic and non-diabetic mice. Data is presented as mean ± SEM with individual values plotted. *P < 0.05 as indicated. Dose (mg/kg) of Bay 41 and Bay 60 indicated in brackets. [file Image1.tif]

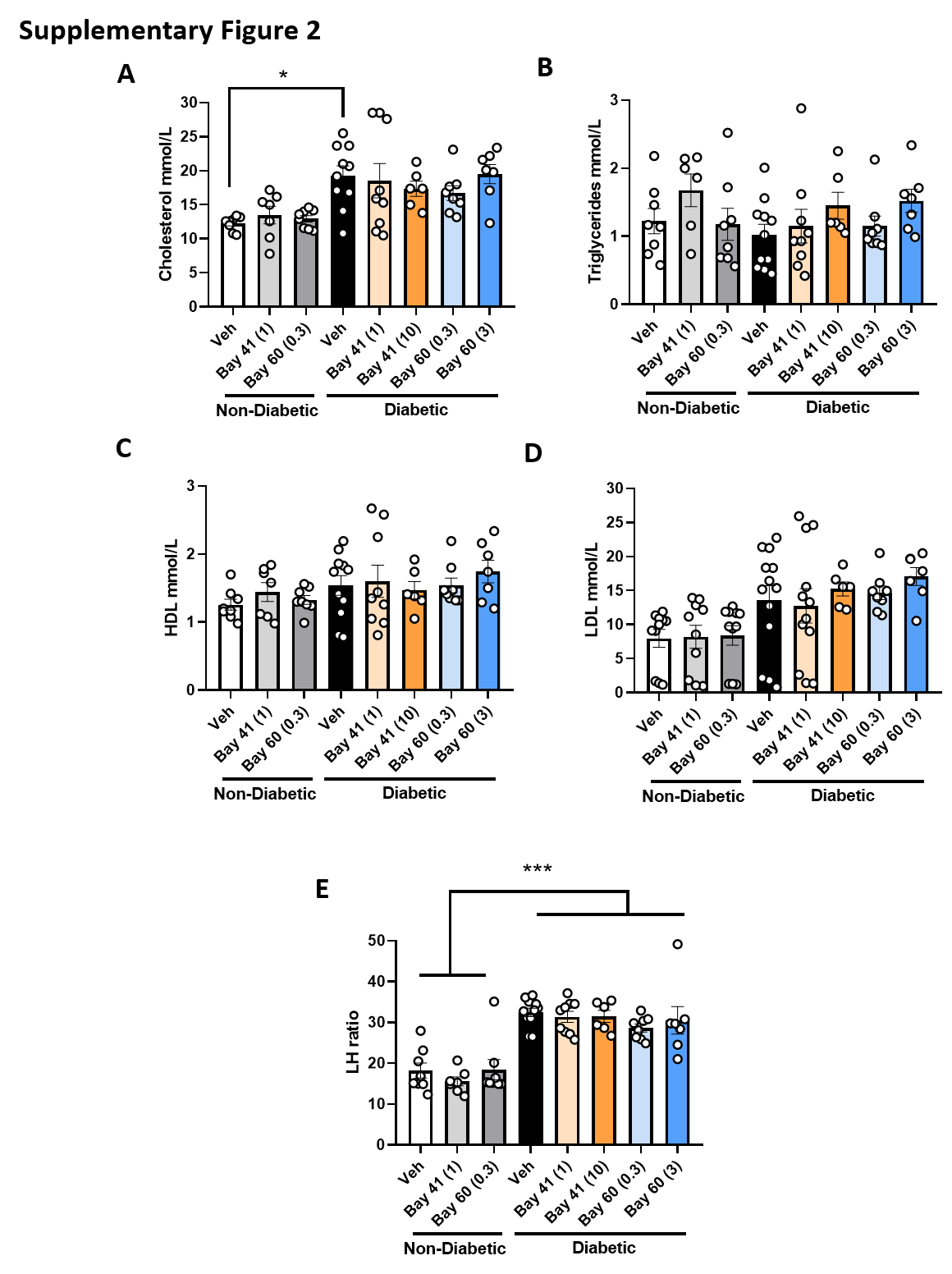

Supplement: Supplementary Figure S2 — Lipid parameters at study end-point (20 weeks) assessed via HPLC. Data is presented as mean ± SEM, with individual values plotted. *P < 0.05 and ***P < 0.001 as indicated. n = 6-13 per group. Dose (mg/kg) of Bay41 and Bay60 indicated in brackets. [file Image2.tif]

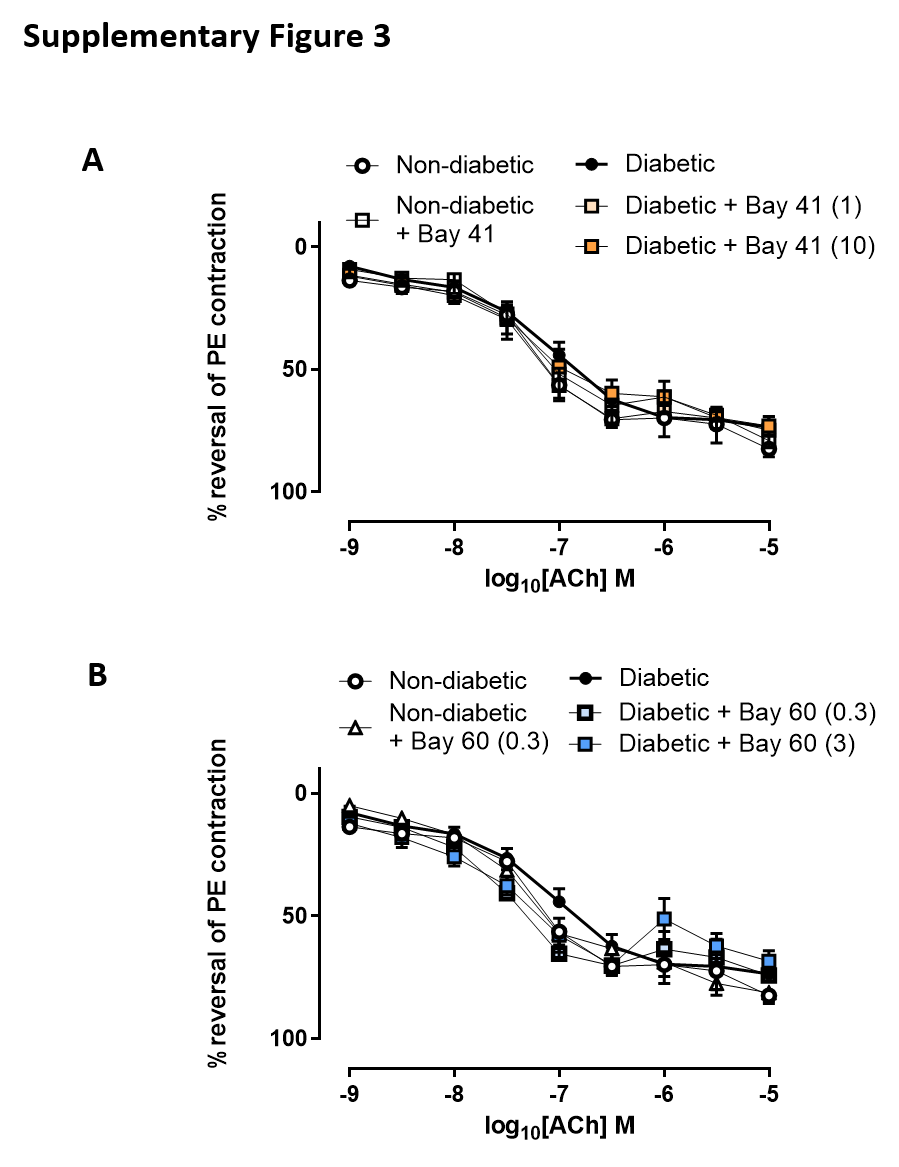

Supplement: Supplementary Figure S3 — (A,B) Vascular relaxation in response to increasing doses of Acetylcholine (Ach) in aortic vessels treated with (A) Bay 41 and (B) Bay 60. Data is presented as mean ± SEM. Dose (mg/kg) of Bay 41 and Bay 60 indicated in brackets. [file Image3.tif]
